# Association between Tumor Necrosis Factor- α-308 G/A Polymorphism and Multiple Sclerosis: A Systematic Review and Meta-Analysis

**Published:** 2014-01

**Authors:** Hamidreza Tolide-ie, Hamid Reza Tabatabaee, Eskandar Kamali-Sarvestani

**Affiliations:** 1 Faculty of Health, Gonabad University of Medical Sciences, Gonabad, Iran;; 2 Department of Epidemiology, School of Health and Nutrition, Shiraz University of Medical Sciences, Shiraz, Iran;; 3 Shiraz Autoimmune Diseases Research Center, Nemazee Hospital, Shiraz University of Medical Sciences, Shiraz, Iran

**Keywords:** Multiple sclerosis, Systematic review, Meta-analysis, Polygenic, Gene

## Abstract

Multiple sclerosis (MS) is a complex polygenic disease in which gene-environment interactions are important. A number of studies have investigated the association between tumor necrosis factor-α (TNF-α) -308 G/A polymorphism (substitution G→A, designated as TNF1 and TNF2) and MS susceptibility in different populations, but the results of individual studies have been inconsistent. Therefore, performing a systematic review and meta-analysis of the published studies is desirable. We sought to quantitatively summarize the association between TNF-α-308 G/A polymorphism and MS. The Medline and Scopus databases were searched to identify potentially relevant case-control studies published in English journals up to January 2010. A meta-analysis of these studies was performed. Summary odds ratios (ORs) and 95% confidence intervals (CIs) were calculated under fixed and random effects models. Twenty-one eligible studies, comprising 2880 patients with MS and 3579 controls, were included in the meta-analysis. The overall pooled ORs (95%CI) for TNF2 versus TNF1 and TNF2 carriers (2/2+2/1) versus non-carriers (1/1) were 1.02 (0.86-1.21) and 0.99 (0.8-1.24), respectively. In the European populations, the pooled ORs (95%CI) for TNF 2/1 versus 1/1 were 0.85 (0.73-0.98), which was statistically significant. However, the other results did not support this finding. The pooled ORs (95%CI) for TNF 2/1 versus 1/1 and TNF 2/2 versus 2/1 were not statistically significant in the overall population. In addition, the pooled ORs for TNF2/2 versus TNF2/1+1/1 and TNF2/2 versus TNF1/1 were not statistically significant. Our meta-analysis does not support the role of TNF-α -308 G/A polymorphism in developing MS.

## Introduction

Multiple sclerosis (MS) is a chronic inflammatory demyelinating disease of the central nervous system. The etiology of MS is complicated, and both environmental and genetic factors play important roles in the pathogenesis of the disease.^1^^,^^2^ Several genes have been investigated, but studies on association with human leukocyte antigen (HLA) have been the most consistent.^2^^-^^4^ Tumor necrosis factor-α (TNF-α) is a pro-inflammatory cytokine, secreted by activated macrophages, with a wide range of biological activities, including induction of tumor regression, fever, cachexia, shock, and cellular immune responses.^5^ High levels of TNF-α have been found in the blood and cerebrospinal fluid (CSF) of MS patients.^6^ TNF-α gene is located on chromosome 6, within the class Ш region of HLA.^7^ A single-nucleotide polymorphism (SNP) at position -308 in the TNF-α gene promoter, defined as TNF1 (-308G) and TNF2 (-308A), has been identified,^8^^,^^9^ in which the less common TNF2 allele is associated with a high production of TNF-α.^10^^,^^11^

A large number of case-control studies have been conducted to investigate the association between TNF-α-308 G/A polymorphism and MS in different populations. However, the results of the individual studies are conflicting, inconsistent, and inconclusive.^12^^-^^32^ Because of small sample sizes in most of these studies, they lacked enough power to detect the probable relationship between this SNP and MS. Since no quantitative summarization of evidence has been performed to date and in order to do a well-powered study in this regard, we conducted a systematic review to find all relevant published studies and performed a meta-analysis to quantitatively summarize the evidence for such a relationship.

## Methods

*Search Strategy*

The Medline (using PubMed) and Scopus databases (last updated search being 1 January 2010) were searched to identify potentially relevant case-control studies. The following keywords were used: polymorphism; multiple sclerosis; and tumor necrosis factor. To find any additional published studies not found by computer search, the reference lists of review articles and all retrieved articles were searched manually at the same time. If more than one article was published by the same author(s) using the same participants, the study that comprised the most individuals or/and had more complementary information was selected. When the written information was insufficient, efforts were made to contact the investigators so as to obtain the needed information. If a reply was not forthcoming or when the contact was impossible, the study was excluded from the meta-analysis. 

The title and abstract of all potentially relevant articles were reviewed to determine their relevance. Additionally, full articles were reviewed if the title and abstract were ambiguous. All the searches were conducted independently by three reviewers and disagreements about the inclusion of a study were resolved by consensus.

*Inclusion and Exclusion Criteria*

The following criteria were used to include studies in the meta-analysis: the study design had to be case-control; the outcome had to be MS; there had to be at least two comparison groups (MS vs. control groups); the number of MS cases and controls and also the frequency of genotypes in both groups had to be identified; and participants could be of any age. English articles and also articles of other languages which had English abstracts with sufficient information (one article) were included in the meta-analysis. Major reasons for the exclusion of studies were: 1) lack of a control group; 2) basing the design on family or sibling pairs; 3) duplication; and 4) not reporting usable data (despite correspondence with authors). 

*Assessment of Study Quality*

Quality assessment was conducted by two investigators using the Little criteria^33^ for genetic studies and the Lichtenstein criteria^34^ for case-control studies. A number of those criteria were: 1) Do the controls and cases come from the same population; 2) Is the same sample used in both groups (e.g. blood); 3) Is there any ethnic matching between the groups?; and 4) Are the methods of genotyping in both groups the same? Subjective assessment was avoided by refraining from the generation of an overall quality score; instead, these criteria were utilized to rank the studies and they are illustrated in tables and forest plots according to their quality ranks. The quality assessors were blinded to the authors, journals, and results of the studies.

*Data Extraction*

Data were extracted from each study independently by two reviewers using a predefined form. To increase reliability and decrease probable biases in data extraction, the following actions were performed:

Before starting, the reviewers had an orientation meeting about how to enter the data or transform some indices. When there was a difference between the reports in the abstracts and full texts, the latter was chosen. Before the confirmation of the final form, a pilot extraction was performed on a number of articles and defects of forms were modified by consensus.

*Statistical Analysis and Heterogeneity Assessment*

Summary odds ratios (ORs) and 95% confidence intervals were calculated from the raw data of the selected studies. For summarizing ORs, the Mantel-Haenzel method based on the fixed effects model was used when there was no heterogeneity between the studies. Otherwise, the DerSimonian and Laird method based on the random effects model was employed. A P value smaller than 0.05 was considered statistically significant. Heterogeneity among the studies was assessed via the x^ 2 ^-based Q test, and a P value smaller than 0.1 was considered statistically significant in the Q test because of its low power. Visual assessment of heterogeneity was illustrated by the Galbraith plot. Subgroup analysis was also conducted only in the European studies, because the number of studies in the other regions was not sufficient.

The Begg rank correlation^35^and the Egger weighted regression methods^36^ were used to statistically assess publication bias. A P value smaller than 0.05 was considered statistically significant for publication bias tests. The funnel plot was also drawn upon for the visual assessment of publication bias. (Asymmetry shows the probable publication bias.) Statistical analysis was performed using STATA 9.0 (Stata Corp., College Station, TX, USA).

## Results

*Characteristics of Included Studies*

**Figure 1:**
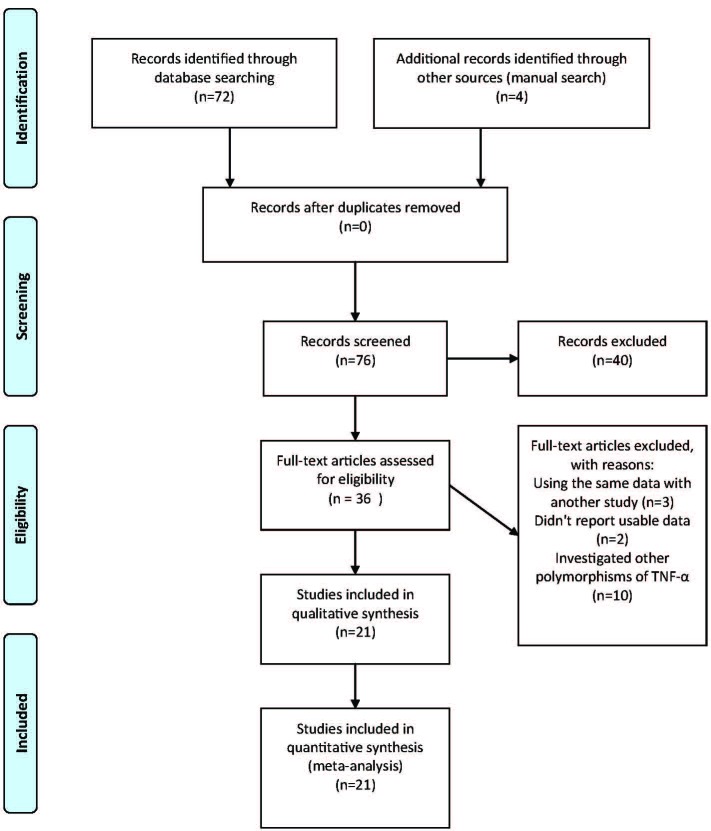
Flow diagram of study results reviewed in our systematic review (n=76).

In the first step, 72 papers were identified. Our manual search of the references identified 4 more articles. However, after the screening stage of the titles and abstracts, forty were excluded because they were either review articles, animal studies, or irrelevant to our study. Full texts of 35 potentially relevant studies and an English abstract of a Chinese article were retrieved and reviewed (totally, 36 studies). From them, 15 were excluded for the following reasons: three articles were duplicates;^37^^-^^39^ two did not report usable data;^40^^,^^41^ and 10 investigated other polymorphisms of TNF-α rather than TNF-α-308.^5^^,^^42^^-^^50^ Finally, twenty-one case-control studies, which comprised 2880 MS cases and 3579 controls, were included in the study.^12^^-^^32^ The selected characteristics of the 21 case-control studies included in this meta-analysis are summarized in table 1 (figure 1).

**Table1:** Characteristics of the 21 studies included in the meta-analysis of tumor necrosis factor-α (TNF-α) -308 polymorphism and multiple sclerosis (MS)*

**Study (year) **	**Source of cases (Number) **	**Source of controls (Number) **
Drulovic (2003)	Belgrade, Serbia (143)	Belgrade, Ethnically matched blood donors (123)
De Jong (2002)	Netherlands, Amsterdam (109) Leiden (50)	Ethnically-matched Dutch organ donors (273)
Ristic (2007)	Croatia (175) Slovenia (163)	Healthy blood donors matched for age, sex, and ethnicity (460)
Fernandes-Filho (2002)	Norway (133)	Healthy controls, same region (148)
Kamali-Sarvestani (2007)	Iran, Shiraz (270)	Healthy volunteers matched for ethnicity and sex (439)
Favorova (2006)	Russia, Moscow (223)	Healthy controls, ethnically matched, free of internal and neurological diseases (222)
Sarial (2008)	Iran, Tehran MS Society (99)	Tehran, random blood samples from Iranian Blood Transfusion Organization (137)
Bing He (1995)	Sweden (93)	Healthy blood donors, ethnically matched (95)
Mihailova (2005)	Bulgaria (55)	Healthy blood donors, ethnically matched (86)
Fernandez Arquero (1999)	Spain (238)	Spain, Madrid (324)
Kirk (1997)	Northern Ireland (189)	Healthy blood donors (106), normal spouses of individuals with single-gene disorder (100), ethnically matched
Forte (2006)	Italy, West Sicily (91)	Healthy controls matched for age, sex, and region(220)
Lucotte (2000)	France (74)	Healthy controls matched for age, sex, and ethnicity (75)
Dong (2006)	South China (68)	South China, ethnically matched (106)
Huizinga (1997)	Nursing home, Belgium (57) and outpatient clinic, Netherlands (98)	Healthy Dutch controls (186)
Mycko (1998)	Poland (53)	Poland, ethnically matched (81)
Braun (1996)	Germany (50)	Healthy controls, ethnically matched (22)
Anlar (2001)	Turkey (24)	DNA bank, Turkey (93)
Wirz (2004)	Italy, Sardinia (32)	Sardinia, healthy controls (35)
Wingerchuk (1997)	US, prevalence cohort of MS in Olmsted county, Mayo Clinic (110)	Patients of other diseases in Mayo Clinic, matched for age, sex, and ethnicity (110)
Maurer (1999)	Germany (283)	Germany, patients of amyotrophic lateral sclerosis (72) and stroke (66)

*Studies were ranked based on their quality.

*Association between Alleles and Genotypes of TNF-α-308 and MS*

**Figure 2:**
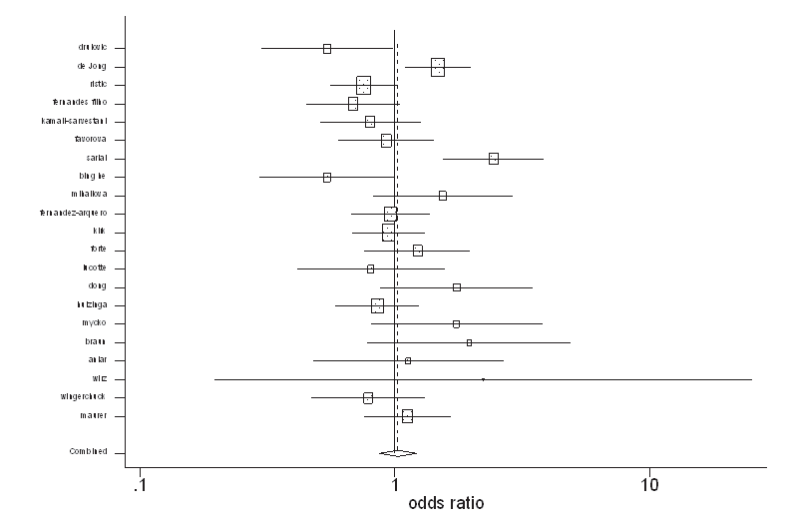
The figure demonstrates the pooled odds ratios and 95% confidence intervals for multiple sclerosis when comparing TNF2 allele with TNF1 allele. The studies are listed based on quality ranking.

**Figure 3:**
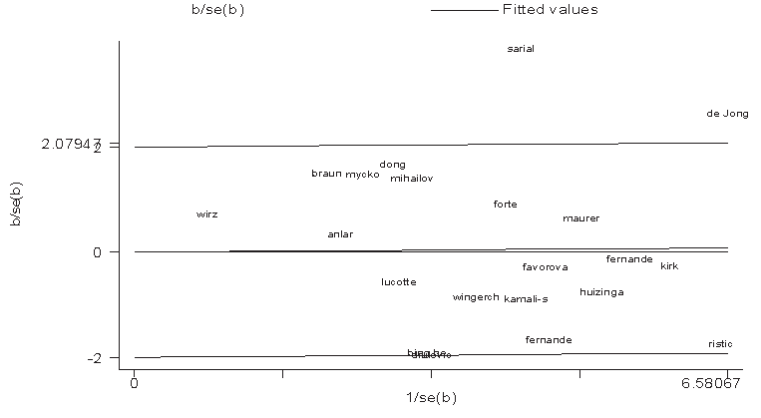
This figures illustrates the Galbraith plot of heterogeneity among the studies in our meta-analysis of tumor necrosis factor-α (TNF-α) -308 gene polymorphism and multiple sclerosis association (TNF2 vs. TNF1 alleles).

**Figure 4:**
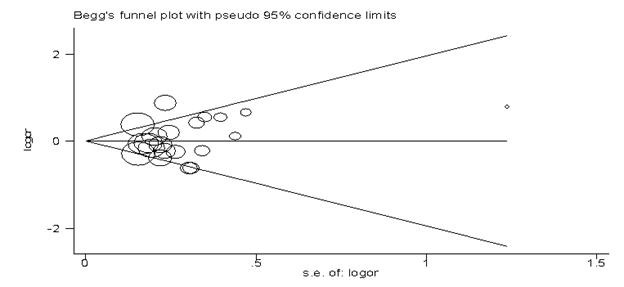
This figure depicted the Begg funnel plot of publication bias in our meta-analysis of tumor necrosis factor-α (TNF-α) -308 gene polymorphism and multiple sclerosis association (TNF2 vs. TNF1 alleles).

The pooled ORs and 95% CIs in both overall and subgroup populations are depicted in table 2. From the meta-analysis, an association between MS and TNF2 allele was not found in the overall (figure 2) and European populations. The Galbraith plot of heterogeneity shows that the Sarial and De Jong studies were the leading causes of heterogeneity between the studies (figure 3). What is more, the Begg and Egger tests and also the funnel plot revealed that there was no significant publication bias in this meta-analysis (table 2, figure 4). The pooled ORs for TNF2/2+2/1 (TNF2+) versus TNF 1/1 (TNF2-) were not significant in the overall and European publications. The pooled ORs for TNF2/1 versus 1/1 were not statistically significant in the overall population, but this association was significant in European studies (OR=0.84, 95% CI: 0.73-0.98). The pooled ORs for TNF2/2 versus TNF 2/1+1/1 and also for TNF2/2 versus TNF 1/1 were not significant in all the comparisons. In addition, the pooled ORs for TNF 2/2 versus TNF 2/1 were not significant either in the overall or in the European publications.

**Table 2:** Meta-analysis of tumor necrosis factor-α (TNF-α) -308 gene polymorphism and multiple sclerosis association

**Comparisons**	**Population**	**N**	**Test of association**		**Test of homogeneity**		**Publication bias**	
			**OR(95%CI) **	**P**	**Q**	**P†**	**Begg (P)**	**Egger (p)**
TNF2 versus TNF1	Total	21	1.02(0.86-1.21)	0.76	48.4	<0.001	0.27	0.51
	European	17	0.97(0.83-1.14)		29.3	0.02		
TNF2+versus TNF2-	Total	18	0.99(0.8-1.24)	0.98	42.4	0.001	0.075	0.15
	European	14	0.86(0.75-1)	0.056	17.6	0.17		
TNF2.1 versus TNF1.1	Total	18	0.97(0.78-1.2)	0.78	39.5	0.001	0.12	0.23
	European	14	0.84 (0.73-0.98)	0.03*	15.46	0.27		
TNF2.2 versus TNF2.1+1.1	Total	15	1.11(0.73-1.71)	0.6	14.3	0.42	1	0.38
	European	13	1.12(0.72-1.75)	0.59	10.7	0.46		
TNF2.2 versus TNF2.1	Total	15	1.36(0.9-2.05)	0.14	12.3	0.58	0.76	0.69
	European	13	1.36(0.88-2.1)	0.16	9.3	0.59		
TNF2.2 versus TNF1.1	Total	15	1.13(0.76-1.68)	0.52	15.2	0.36	0.8	0.3
	European	13	1.07(0.68-1.67)	0.73	11.4	0.41		

OR, odds ratio; CI, confidence interval; *Statistically significant; †P<0.1 is considered statistically significant for Q statistics

## Discussion

MS is a complex polygenic disease, and the dissection of its genetic background is very complicated because of the combinatorial possibilities of gene-gene interactions. Our meta-analysis reveals that the inheritance of TNF2 allele does not change the risk of MS. In the meta-analysis of genotypes, although we witnessed that 2/1 heterozygote decreased the risk of MS in comparison with 1/1 homozygote in the European publications, other comparisons did not support this result. For instance, considering the dose-response correlation, it was expected that 2/2 homozygote would exhibit a stronger negative association than 2/1 heterozygote with MS, but we did not find these results in our different comparisons. 

On the other hand, some studies have suggested that TNF2 allele is associated with a high production of TNF-α^10^^,^^11^ and that the level of TNF-alpha in the CSF correlates with the severity and progression of MS.^6^ It is, therefore, expected that the carriers of TNF2 alleles have more chance of developing MS than TNF1 carriers. Be that as it my, our meta-analysis did not support this interpretation.

It seems that other polymorphisms in different positions of TNF-α, other cytokines, and also their interaction should be taken into account in the study of MS susceptibility. Recently, some genome-wide association studies (GWAS) were performed by analyzing a large number of SNPs, simultaneously, based on chip technology and demonstrated no significant relationship between TNF-α-308 gene polymorphism and MS,^51^^-^^54^ which is consistent with our findings. It is crystal clear that these kinds of studies that consider different gene variations at the same time and also studies that analyze gene/gene and gene/environment interactions would be more reliable to reach the concise results about the exact contribution of genes in this complex disease.

There were some limitations in this meta-analysis. Firstly, in some comparisons, the pooled ORs were obtained from heterogeneous studies. Secondly, only published studies were included in this meta-analysis; consequently, publication bias may have occurred, although the funnel plots and statistical tests did not show it in our meta-analysis. Thirdly, assessment and quality ranking of the studies was according to their reports and also was very subjective, precluding us from considering this ranking as a definite criterion. Finally, meta-analysis is a retrospective research that is subject to methodological deficiencies and potential biases in the studies included.  

## Conclusion

Our meta-analysis does not support the role of TNF-α -308 G/A polymorphism in developing MS.

## References

[1] Ebers GC, Sadovnick AD (1994). The role of genetic factors in multiple sclerosis susceptibility. J Neuroimmunol.

[2] Oksenberg JR, Begovich AB, Erlich HA, Steinman L (1993). Genetic factors in multiple sclerosis. JAMA.

[3] Reboul J, Mertens C, Levillayer F, Eichenbaum-Voline S, Vilkoren T, Cournu I (2000). Cytokines in genetic susceptibility to multiple sclerosis: a candidate gene approach. French Multiple Sclerosis Genetics Group. J Neuroimmunol.

[4] Kantarci OH, de Andrade, Weinshenker BG (2002). Identifying disease modifying genes in multiple sclerosis. J Neuroimmunol.

[5] Fugger L, Morling N, Sandberg-Wollheim M, Ryder LP, Svejgaard A (1990). Tumor necrosis factor alpha gene polymorphism in multiple sclerosis and optic neuritis. J Neuroimmunol.

[6] Sharief MK, Hentges R (1991). Association between tumor necrosis factor-alpha and disease progression in patients with multiple sclerosis. N Engl J Med.

[7] Lu Z, Chen L, Li H, Zhao Y, Lin L (2008). Effect of the polymorphism of tumor necrosis factor-alpha-308 G/A gene promoter on the susceptibility to ulcerative colitis: a meta-analysis. Digestion.

[8] Wilson AG, di Giovine, Blakemore AI, Duff GW (1992). Single base polymorphism in the human tumour necrosis factor alpha (TNF alpha) gene detectable by NcoI restriction of PCR product. Hum Mol Genet.

[9] Abraham LJ, Kroeger KM (1999). Impact of the -308 TNF promoter polymorphism on the transcriptional regulation of the TNF gene: relevance to disease. J Leukoc Biol.

[10] Pociot F, Briant L, Jongeneel CV, Mölvig J, Worsaae H, Abbal M (1993). Association of tumor necrosis factor (TNF) and class II major histocompatibility complex alleles with the secretion of TNF-alpha and TNF-beta by human mononuclear cells: a possible link to insulin-dependent diabetes mellitus. Eur J Immunol.

[11] Candore G, Cigna D, Gervasi F, Colucci AT, Modica MA, Caruso C (1994). In vitro cytokine production by HLA-B8,DR3 positive subjects. Autoimmunity.

[12] Drulović J, Popadić D, Mesaros S, Dujmović I, Cvetković I, Miljković D (2003). Decreased frequency of the tumor necrosis factor alpha -308 allele in Serbian patients with multiple sclerosis. Eur Neurol.

[13] Kamali-Sarvestani E, Nikseresht A, Aflaki E, Sarvari J, Gharesi-Fard B (2007). TNF-alpha, TNF-beta and IL-4 gene polymorphisms in Iranian patients with multiple sclerosis. Acta Neurol Scand.

[14] de Jong, Huizinga TW, Zanelli E, Giphart MJ, Bollen EL, Uitdehaag BM (2002). Evidence for additional genetic risk indicators of relapse-onset MS within the HLA region. Neurology.

[15] He B, Navikas V, Lundahl J, Söderström M, Hillert J (1995). Tumor necrosis factor alpha-308 alleles in multiple sclerosis and optic neuritis. J Neuroimmunol.

[16] Braun N, Michel U, Ernst BP, Metzner R, Bitsch A, Weber F (1996). Gene polymorphism at position -308 of the tumor-necrosis-factor-alpha (TNF-alpha) in multiple sclerosis and it’s influence on the regulation of TNF-alpha production. Neurosci Lett.

[17] Kirk CW, Droogan AG, Hawkins SA, McMillan SA, Nevin NC, Graham CA (1997). Tumour necrosis factor microsatellites show association with multiple sclerosis. J Neurol Sci.

[18] Huizinga TW, Westendorp RG, Bollen EL, Keijsers V, Brinkman BM, Langermans JA (1997). TNF-alpha promoter polymorphisms, production and susceptibility to multiple sclerosis in different groups of patients. J Neuroimmunol.

[19] Wingerchuk D, Liu Q, Sobell J, Sommer S, Weinshenker BG (1997). A population-based case-control study of the tumor necrosis factor alpha-308 polymorphism in multiple sclerosis. Neurology.

[20] Mycko M, Kowalski W, Kwinkowski M, Buenafe AC, Szymanska B, Tronczynska E (1998). Multiple sclerosis: the frequency of allelic forms of tumor necrosis factor and lymphotoxin-alpha. J Neuroimmunol.

[21] Fernandez-Arquero M, Arroyo R, Rubio A, Martin C, Vigil P, Conejero L (1999). Primary association of a TNF gene polymorphism with susceptibility to multiple sclerosis. Neurology.

[22] Mäurer M, Kruse N, Giess R, Kyriallis K, Toyka KV, Rieckmann P (1999). Gene polymorphism at position -308 of the tumor necrosis factor alpha promotor is not associated with disease progression in multiple sclerosis patients. J Neurol.

[23] Lucotte G, Bathelier C, Mercier G (2000). TNF-alpha polymorphisms in multiple sclerosis: no association with -238 and -308 promoter alleles, but the microsatellite allele a11 is associated with the disease in French patients. Mult Scler.

[24] Anlar B, Alikaşifoglu M, Köse G, Güven A, Gürer Y, Yakut A (2001). Tumor necrosis factor-alpha gene polymorphisms in children with multiple sclerosis. Neuropediatrics.

[25] Fernandes Filho, Vedeler CA, Myhr KM, Nyland H, Pandey JP (2002). TNF-alpha and -beta gene polymorphisms in multiple sclerosis: a highly significant role for determinants in the first intron of the TNF-beta gene. Autoimmunity.

[26] Mihailova S, Ivanova M, Mihaylova A, Quin L, Mikova O, Naumova E (2005). Pro- and anti-inflammatory cytokine gene polymorphism profiles in Bulgarian multiple sclerosis patients. J Neuroimmunol.

[27] Ristić S, Lovrecić L, Starcević-Cizmarević N, Brajenović-Milić B, Sega Jazbec S, Sepcić J (2007). Tumor necrosis factor-alpha-308 gene polymorphism in Croatian and Slovenian multiple sclerosis patients. Eur Neurol.

[28] Favorova OO, Favorov AV, Boiko AN, Andreewski TV, Sudomoina MA, Alekseenkov AD (2006). Three allele combinations associated with multiple sclerosis. BMC Med Genet.

[29] Sarial S, Shokrgozar MA, Amirzargar A, Shokri F, Radfar J, Zohrevand P (2008). IL-1, IL-1R and TNFalpha gene polymorphisms in Iranian patients with multiple sclerosis. Iran J Allergy Asthma Immunol.

[30] Forte GI, Ragonese P, Salemi G, Scola L, Candore G, D’Amelio M (2006). Search for genetic factors associated with susceptibility to multiple sclerosis. Ann N Y Acad Sci.

[31] Wirz SA, Morale MC, Marchetti B, Barr AM, Sotgiu S, Rosati G (2004). High frequency of TNF alleles -238A and -376A in individuals from northern Sardinia. Cytokine.

[32] Dong YX, Xu ZR, Lin PY (2006). [Association among serous and cerebrospinal fluid TNF-alpha level, gene polymorphisms of TNF-alpha and multiple sclerosis in Han nationality of southern China]. Zhonghua Yi Xue Yi Chuan Xue Za Zhi.

[33] Little J, Bradley L, Bray MS, Clyne M, Dorman J, Ellsworth DL (2002). Reporting, appraising, and integrating data on genotype prevalence and gene-disease associations. Am J Epidemiol.

[34] Lichtenstein MJ, Mulrow CD, Elwood PC (1987). Guidelines for reading case-control studies. J Chronic Dis.

[35] Begg CB, Mazumdar M (1994). Operating characteristics of a rank correlation test for publication bias. Biometrics.

[36] Egger M, Davey Smith, Schneider M, Minder C (1997). Bias in meta-analysis detected by a simple, graphical test. BMJ.

[37] Amirzargar A, Khosravi F, Dianat S, Hushmand F, Maryousef P, Foroushani AR (2007). Profile of cytokine gene polymorphisms in Iranian multiple sclerosis patients. Mult Scler.

[38] Alexeenkov AD, Sudomoina MA, Boiko AN, Deomina TL, Gusev EI, Favorova OO (1999). Two single-base polymorphisms in the human tumor necrosis factor locus of multiple sclerosis patients from the Russian population: NcoI-RFLP in the first intron of the human lymphotoxin alpha gene correlates with the disease. Mol Biol.

[39] Weinshenker BG, Wingerchuk DM, Liu Q, Bissonet AS, Schaid DJ, Sommer SS (1997). Genetic variation in the tumor necrosis factor alpha gene and the outcome of multiple sclerosis. Neurology.

[40] Epplen C, Jäckel S, Santos EJ, D’Souza M, Poehlau D, Dotzauer B (1997). Genetic predisposition to multiple sclerosis as revealed by immunoprinting. Ann Neurol.

[41] Ma JJ, Nishimura M, Mine H, Saji H, Ohta M, Saida K (1998). HLA-DRB1 and tumor necrosis factor gene polymorphisms in Japanese patients with multiple sclerosis. J Neuroimmunol.

[42] McDonnell GV, Kirk CW, Middleton D, Droogan AG, Hawkins SA, Patterson CC (1999). Genetic association studies of tumour necrosis factor alpha and beta and tumour necrosis factor receptor 1 and 2 polymorphisms across the clinical spectrum of multiple sclerosis. J Neurol.

[43] Palacio LG, Rivera D, Builes JJ, Jiménez ME, Salgar M, Anaya JM (2002). Multiple sclerosis in the tropics: genetic association to STR’s loci spanning the HLA and TNF. Mult Scler.

[44] Sandberg-Wollheim M, Ciusani E, Salmaggi A, Pociot F (1995). An evaluation of tumor necrosis factor microsatellite alleles in genetic susceptibility to multiple sclerosis. Mult Scler.

[45] Allcock RJ, de la, Fernandez-Arquero M, Vigil P, Conejero L, Arroyo R (1999). Susceptibility to multiple sclerosis mediated by HLA-DRB1 is influenced by a second gene telomeric of the TNF cluster. Hum Immunol.

[46] Roth MP, Nogueira L, Coppin H, Clanet M, Clayton J, Cambon-Thomsen A (1994). Tumor necrosis factor polymorphism in multiple sclerosis: no additional association independent of HLA. J Neuroimmunol.

[47] Armstrong MA, McDonnell GV, Graham CA, Kirk CW, Droogan AG, Hawkins SA (1999). Relationship between tumour necrosis factor-alpha (TNFalpha) production and a specific multiple sclerosis (MS) associated TNF gene haplotype. Mult Scler.

[48] Weinshenker BG, Hebrink DD, Atkinson E, Kantarci OH (2001). Association of a tumor necrosis factor alpha polymorphism with MS susceptibility. Neurology.

[49] Kikuchi S, Miyagishi R, Fukazawa T, Yabe I, Miyazaki Y, Sasaki H (2005). TNF-related apoptosis inducing ligand (TRAIL) gene polymorphism in Japanese patients with multiple sclerosis. J Neuroimmunol.

[50] Trojano M, Liguori M, De Robertis, Stella A, Guanti G, Avolio C (1999). Comparison of clinical and demographic features between affected pairs of Italian multiple sclerosis multiplex families; relation to tumour necrosis factor genomic polymorphisms. J Neurol Sci.

[51] Australia and (2009). Genome-wide association study identifies new multiple sclerosis susceptibility loci on chromosomes 12 and 20. Nat Genet.

[52] Comabella M, Craig DW, Camiña-Tato M, Morcillo C, Lopez C, Navarro A (2008). Identification of a novel risk locus for multiple sclerosis at 13q31.3 by a pooled genome-wide scan of 500,000 single nucleotide polymorphisms. PLoS One.

[53] Aulchenko YS, Hoppenbrouwers IA, Ramagopalan SV, Broer L, Jafari N, Hillert J (2008). Genetic variation in the KIF1B locus influences susceptibility to multiple sclerosis. Nat Genet.

[54] Jakkula E, Leppä V, Sulonen AM, Varilo T, Kallio S, Kemppinen A (2010). Genome-wide association study in a high-risk isolate for multiple sclerosis reveals associated variants in STAT3 gene. Am J Hum Genet.

